# Crystal structure of (2-methyl-1-phenyl­sulfon­yl-1*H*-indol-3-yl)(phen­yl)methanone

**DOI:** 10.1107/S2056989014028059

**Published:** 2015-01-03

**Authors:** M. Umadevi, V. Saravanan, R. Yamuna, A. K. Mohanakrishnan, G. Chakkaravarthi

**Affiliations:** a Research and Development Centre, Bharathiyar University, Coimbatore 641 046, India; b Department of Chemistry, Pallavan College of Engineering, Kanchipuram 631 502, India; c Department of Organic Chemistry, University of Madras, Guindy Campus, Chennai 600 025, India; d Department of Sciences, Chemistry and Materials Research Lab, Amrita Vishwa Vidyapeetham University, Ettimadai, Coimbatore 641 112, India; e Department of Physics, CPCL Polytechnic College, Chennai 600 068, India

**Keywords:** Indole derivative, crystal structure, C—H⋯O hydrogen bonds

## Abstract

In the title compound, C_22_H_17_NO_3_S, the sulfonyl-bound phenyl ring is almost orthogonal to the indole ring system, making a dihedral angle of 84.89 (7)°. The carbonyl-bound phenyl ring forms a dihedral angle of 57.32 (5)° with the indole ring system. The two phenyl rings are inclined at 52.68 (7)°. The S atom has a distorted tetra­hedral configuration. In the crystal, weak C—H⋯O inter­actions link the mol­ecules, forming a helical chain along the *b*-axis direction.

## Abstract 2

In the title compound, the indole ring system makes the dihedral angles of 84.89 (7) and 57.32 (5)° with the phenyl rings. In the crystal, mol­ecules are linked by C—H⋯O hydrogen bonds.

## Chemical context   

In a continuation of our studies on indole derivatives, which possess various biological activities such as anti­hepatitis B virus (Chai *et al.*, 2006 ▸) and anti­bacterial (Nieto *et al.*, 2005 ▸) *etc*, we herein report the synthesis and the crystal structure of the title compound, (I).
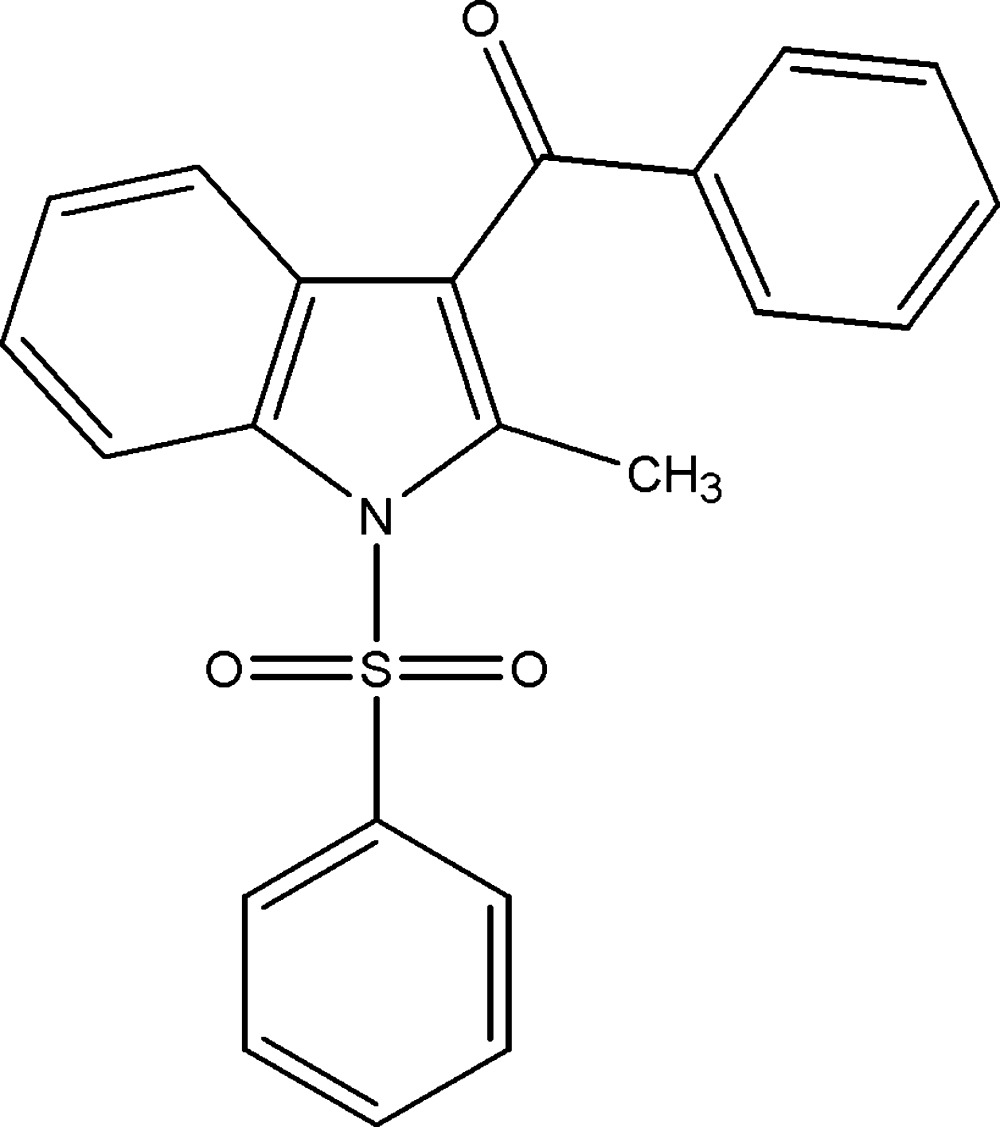


## Structural commentary   

**Figure 1:**
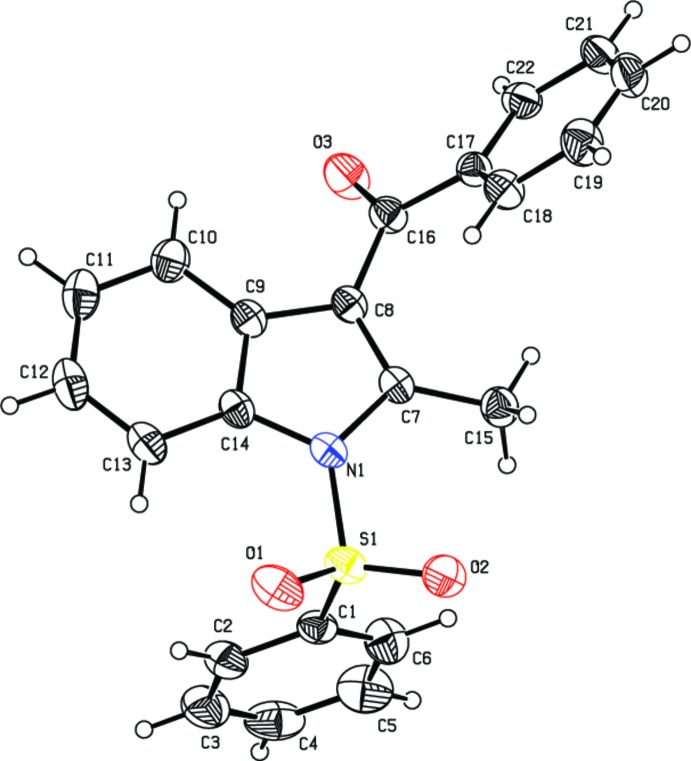
The mol­ecular structure of the title compound, with atom labels and 30% probability displacement ellipsoids for non-H atoms.

The mol­ecular structure of the title compound is shown in Fig. 1 ▸. The sulfonyl-bound phenyl ring (C1–C6) is almost orthogonal to the indole ring system (N1/C7–C14), making a dihedral angle of 84.89 (7)°. The carbonyl-bound phenyl ring (C17–C22) forms a dihedral angle of 57.32 (5)° with the indole ring system. The two phenyl rings are inclined at an angle of 52.68 (7)°. Atom S1 has a distorted tetra­hedral configuration with angles O1—S1—O2 [119.97 (10)°] and N1—S1—C1 [104.99 (8)°] differing from the ideal tetra­hedral value. As a result of the electron-withdrawing character of the phenyl­sulfonyl group, the bond lengths N1—C7 [1.420 (2) Å] and N1—C14 [1.419 (2) Å] are longer than the mean value of 1.355 (14) Å (Allen *et al.*, 1987 ▸). The geometric parameters of (I) agree well with those in similar reported structures (Chakkaravarthi *et al.*, 2008 ▸, 2009 ▸).

## Supra­molecular features   

**Figure 2:**
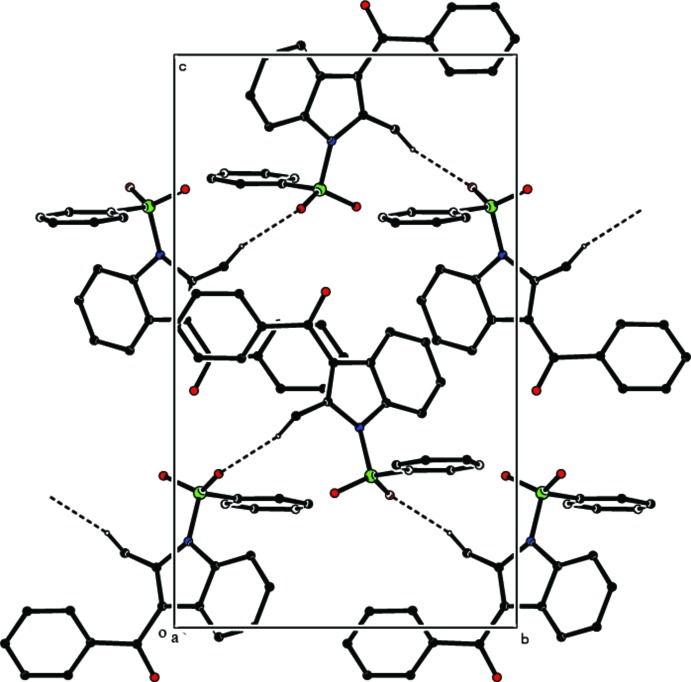
The packing diagram of the title compound, viewed down the *a* axis. Inter­molecular hydrogen bonds are shown as dashed lines. H atoms not involved in hydrogen bonding have been omitted.

In the crystal, weak C—H⋯O inter­actions link the mol­ecules, forming a helical chain along the *b-*axis direction (Table 1 ▸ and Fig. 2 ▸). No significant π–π or C—H⋯π inter­actions are observed.

## Database survey   

A search of the Cambridge Structural Database (Version 5.35, last update May 2014; Groom & Allen, 2014 ▸). indicated 123 compounds having a phenyl­sulfonyl-1*H*-indole moiety. Of these compounds, several similar structures have been reported earlier, *i.e.* ethyl 2-acet­oxy­methyl-1-phenyl­sulfonyl-1*H*-indole-3-carboxyl­ate (Gunasekaran *et al.*, 2009 ▸), 3-iodo-2-methyl-1-phenyl­sulfonyl-1*H*-indole (Ramathilagam *et al.*, 2011 ▸) and 1-(2-bromo­methyl-1-phenyl­sulfonyl-1*H*-indol-3-yl)propan-1-one (Umadevi *et al.*, 2013 ▸). In these structures, the sulfonyl-bound phenyl ring is almost orthogonal to the indole ring system, the dihedral angles of 83.35 (5), 82.84 (9) and 89.91 (11)°, respectively, being are comparable with that in the title compound.

## Synthesis and crystallization   

To a solution of benzoyl chloride (1.55 g, 11.07 mmol) in dry DCM (25 ml), SnCl_4_ (2.88 g, 10.10 mmol) at 273 K was added dropwise. To this, phenyl­sulfonyl-1*H*-indole (2 g, 7.38 mmol) in dry DCM (10 ml) was added dropwise (5 min) and stirred for 30 min at the same temperature. After completion of the reaction (monitored by TLC), it was poured over ice–water (50 ml) and extracted with saturated aqueous NaHCO_3_ (2 × 30 ml) and brine (2 × 30 ml), dried (Na_2_SO_4_) and concentrated under reduced pressure. Then, the crude product was crystallized from methanol to afford single crystals of the title compound suitable for X-ray diffraction.

## Refinement   

Crystal data, data collection and structure refinement details are summarized in Table 2 ▸. H atoms for C_aromatic_ and C_meth­yl_ were positioned geometrically and refined using a riding model, with C—H = 0.93 and 0.97 Å, respectively with *U*_iso_(H) = 1.5*U*_eq_(C) for methyl H atoms and 1.2*U*_eq_(C) for other H atoms.

## Supplementary Material

Crystal structure: contains datablock(s) global, I. DOI: 10.1107/S2056989014028059/is5387sup1.cif

Structure factors: contains datablock(s) I. DOI: 10.1107/S2056989014028059/is5387Isup2.hkl

Supporting information file. DOI: 10.1107/S2056989014028059/is5387Isup3.cml

CCDC reference: 1040926

Additional supporting information:  crystallographic information; 3D view; checkCIF report

## Figures and Tables

**Table 1:** Hydrogen-bond geometry (, )

*D*H*A*	*D*H	H*A*	*D* *A*	*D*H*A*
C15H15*C*O1^i^	0.96	2.59	3.525(3)	165

Symmetry code: (i) 
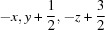
.

**Table 2:** Experimental details

Crystal data	
Chemical formula	C_22_H_17_NO_3_S
*M*_r_	375.43
Crystal system, space group	Orthorhombic, *P*2_1_2_1_2_1_
Temperature (K)	295
*a*, *b*, *c* ()	8.9989(7), 11.0036(9), 18.4209(16)
*V* (^3^)	1824.0(3)
*Z*	4
Radiation type	Mo *K*
(mm^1^)	0.20
Crystal size (mm)	0.28 0.24 0.20

Data collection	
Diffractometer	Bruker APEXII CCD
Absorption correction	Multi-scan (*SADABS*; Sheldrick, 1996 ▸)
*T*_min_, *T*_max_	0.946, 0.961
No. of measured, independent and observed [*I* > 2(*I*)] reflections	26244, 5020, 3493
*R*_int_	0.034
(sin /)_max_ (^1^)	0.708

Refinement	
*R*[*F* ^2^ > 2(*F* ^2^)], *wR*(*F* ^2^), *S*	0.036, 0.091, 1.02
No. of reflections	5020
No. of parameters	246
H-atom treatment	H-atom parameters constrained
_max_, _min_ (e ^3^)	0.17, 0.25
Absolute structure	Flack (1983 ▸), 2109 Friedel pairs
Absolute structure parameter	0.01(7)

Computer programs: *APEX2* and *SAINT* (Bruker, 2004 ▸), *SHELXS97* and *SHELXL97* (Sheldrick, 2008 ▸) and *PLATON* (Spek, 2009 ▸).

## supplementary crystallographic information

### Crystal data

**Table:**

C_22_H_17_NO_3_S	*F*(000) = 784
*M**_r_* = 375.43	*D*_x_ = 1.367 Mg m^−^^3^
Orthorhombic, *P*2_1_2_1_2_1_	Mo *K*α radiation, λ = 0.71073 Å
Hall symbol: P 2ac 2ab	Cell parameters from 812 reflections
*a* = 8.9989 (7) Å	θ = 2.2–30.2°
*b* = 11.0036 (9) Å	µ = 0.20 mm^−^^1^
*c* = 18.4209 (16) Å	*T* = 295 K
*V* = 1824.0 (3) Å^3^	Block, colourless
*Z* = 4	0.28 × 0.24 × 0.20 mm

### Data collection

**Table:**

Bruker APEXII CCD diffractometer	5020 independent reflections
Radiation source: fine-focus sealed tube	3493 reflections with *I* > 2σ(*I*)
Graphite monochromator	*R*_int_ = 0.034
ω and φ scan	θ_max_ = 30.2°, θ_min_ = 2.2°
Absorption correction: multi-scan (*SADABS*; Sheldrick, 1996)	*h* = −12→12
*T*_min_ = 0.946, *T*_max_ = 0.961	*k* = −14→14
26244 measured reflections	*l* = −25→24

### Refinement

**Table:**

Refinement on *F*^2^	Hydrogen site location: inferred from neighbouring sites
Least-squares matrix: full	H-atom parameters constrained
*R*[*F*^2^ > 2σ(*F*^2^)] = 0.036	*w* = 1/[σ^2^(*F*_o_^2^) + (0.0353*P*)^2^ + 0.2764*P*] where *P* = (*F*_o_^2^ + 2*F*_c_^2^)/3
*wR*(*F*^2^) = 0.091	(Δ/σ)_max_ < 0.001
*S* = 1.02	Δρ_max_ = 0.17 e Å^−^^3^
5020 reflections	Δρ_min_ = −0.25 e Å^−^^3^
246 parameters	Extinction correction: *SHELXL97* (Sheldrick, 2008), Fc^*^=kFc[1+0.001xFc^2^λ^3^/sin(2θ)]^-1/4^
0 restraints	Extinction coefficient: 0.0039 (8)
Primary atom site location: structure-invariant direct methods	Absolute structure: Flack (1983), 2109 Friedel pairs
Secondary atom site location: difference Fourier map	Absolute structure parameter: −0.01 (7)

### Special details

**Table:**

Geometry. All esds (except the esd in the dihedral angle between two l.s. planes) are estimated using the full covariance matrix. The cell esds are taken into account individually in the estimation of esds in distances, angles and torsion angles; correlations between esds in cell parameters are only used when they are defined by crystal symmetry. An approximate (isotropic) treatment of cell esds is used for estimating esds involving l.s. planes.
Refinement. Refinement of F^2^ against ALL reflections. The weighted R-factor wR and goodness of fit S are based on F^2^, conventional R-factors R are based on F, with F set to zero for negative F^2^. The threshold expression of F^2^ > 2sigma(F^2^) is used only for calculating R-factors(gt) etc. and is not relevant to the choice of reflections for refinement. R-factors based on F^2^ are statistically about twice as large as those based on F, and R- factors based on ALL data will be even larger.

### Fractional atomic coordinates and isotropic or equivalent isotropic displacement parameters (Å^2^)

**Table:**

	*x*	*y*	*z*	*U*_iso_*/*U*_eq_	
C1	0.2369 (2)	0.81475 (17)	0.72517 (9)	0.0469 (4)	
C2	0.2009 (3)	0.69315 (19)	0.72539 (12)	0.0623 (6)	
H2	0.1029	0.6684	0.7318	0.075*	
C3	0.3119 (4)	0.6089 (2)	0.71605 (14)	0.0779 (8)	
H3	0.2891	0.5265	0.7160	0.093*	
C4	0.4552 (4)	0.6457 (3)	0.70682 (13)	0.0797 (8)	
H4	0.5295	0.5881	0.6999	0.096*	
C5	0.4904 (3)	0.7661 (3)	0.70759 (15)	0.0815 (8)	
H5	0.5889	0.7903	0.7025	0.098*	
C6	0.3812 (3)	0.8512 (2)	0.71586 (13)	0.0658 (6)	
H6	0.4047	0.9335	0.7152	0.079*	
C7	0.1037 (2)	1.05176 (15)	0.60528 (9)	0.0405 (4)	
C8	0.0471 (2)	1.03514 (16)	0.53755 (10)	0.0417 (4)	
C9	−0.0431 (2)	0.92698 (17)	0.53745 (10)	0.0421 (4)	
C10	−0.1247 (2)	0.86956 (18)	0.48385 (12)	0.0566 (5)	
H10	−0.1258	0.8998	0.4367	0.068*	
C11	−0.2046 (3)	0.7663 (2)	0.50152 (15)	0.0667 (6)	
H11	−0.2593	0.7266	0.4659	0.080*	
C12	−0.2040 (3)	0.72186 (19)	0.57128 (15)	0.0651 (6)	
H12	−0.2588	0.6524	0.5819	0.078*	
C13	−0.1248 (2)	0.77715 (17)	0.62583 (13)	0.0549 (5)	
H13	−0.1251	0.7466	0.6729	0.066*	
C14	−0.0439 (2)	0.88084 (16)	0.60774 (11)	0.0429 (4)	
C15	0.2119 (2)	1.14443 (19)	0.63034 (11)	0.0521 (5)	
H15A	0.2445	1.1921	0.5897	0.078*	
H15B	0.2959	1.1047	0.6520	0.078*	
H15C	0.1654	1.1964	0.6655	0.078*	
C16	0.0714 (2)	1.10898 (16)	0.47141 (10)	0.0462 (4)	
C17	0.0621 (2)	1.24405 (16)	0.47428 (10)	0.0425 (4)	
C18	−0.0098 (2)	1.30428 (18)	0.52995 (12)	0.0513 (5)	
H18	−0.0503	1.2605	0.5683	0.062*	
C19	−0.0215 (3)	1.4297 (2)	0.52874 (14)	0.0667 (6)	
H19	−0.0699	1.4700	0.5663	0.080*	
C20	0.0380 (3)	1.4945 (2)	0.47233 (15)	0.0684 (7)	
H20	0.0296	1.5787	0.4716	0.082*	
C21	0.1096 (3)	1.4360 (2)	0.41704 (13)	0.0633 (6)	
H21	0.1511	1.4805	0.3792	0.076*	
C22	0.1204 (2)	1.31043 (19)	0.41723 (11)	0.0512 (5)	
H22	0.1671	1.2707	0.3789	0.061*	
N1	0.04557 (17)	0.95902 (13)	0.65071 (8)	0.0436 (4)	
O1	−0.02677 (18)	0.87038 (14)	0.76860 (8)	0.0700 (4)	
O2	0.16072 (19)	1.03169 (12)	0.76515 (8)	0.0632 (4)	
O3	0.0922 (2)	1.05764 (13)	0.41366 (7)	0.0699 (4)	
S1	0.09803 (6)	0.92484 (4)	0.73525 (3)	0.04934 (14)	

### Atomic displacement parameters (Å^2^)

**Table:**

	*U*^11^	*U*^22^	*U*^33^	*U*^12^	*U*^13^	*U*^23^
C1	0.0585 (12)	0.0484 (10)	0.0339 (9)	0.0054 (9)	0.0015 (9)	0.0024 (9)
C2	0.0712 (14)	0.0513 (12)	0.0644 (14)	0.0075 (11)	−0.0075 (12)	0.0094 (11)
C3	0.103 (2)	0.0516 (14)	0.0789 (17)	0.0201 (14)	−0.0139 (16)	−0.0001 (12)
C4	0.098 (2)	0.0856 (19)	0.0556 (14)	0.0464 (17)	−0.0027 (14)	−0.0015 (13)
C5	0.0655 (17)	0.095 (2)	0.0836 (19)	0.0196 (15)	0.0100 (14)	0.0062 (16)
C6	0.0626 (14)	0.0606 (13)	0.0743 (15)	0.0054 (12)	0.0048 (12)	−0.0017 (11)
C7	0.0405 (9)	0.0364 (9)	0.0448 (9)	0.0027 (8)	0.0077 (8)	0.0009 (7)
C8	0.0453 (10)	0.0362 (9)	0.0436 (10)	0.0040 (8)	0.0065 (8)	−0.0015 (8)
C9	0.0441 (9)	0.0346 (9)	0.0476 (10)	0.0067 (8)	0.0068 (8)	−0.0029 (8)
C10	0.0608 (13)	0.0529 (11)	0.0560 (12)	−0.0004 (11)	0.0006 (11)	−0.0103 (10)
C11	0.0660 (15)	0.0526 (13)	0.0813 (16)	−0.0068 (11)	0.0007 (13)	−0.0177 (12)
C12	0.0555 (14)	0.0402 (11)	0.0995 (19)	−0.0051 (10)	0.0087 (13)	−0.0040 (12)
C13	0.0497 (12)	0.0417 (10)	0.0732 (13)	0.0019 (10)	0.0084 (11)	0.0106 (10)
C14	0.0395 (9)	0.0350 (9)	0.0541 (11)	0.0043 (8)	0.0071 (8)	0.0014 (8)
C15	0.0556 (12)	0.0503 (11)	0.0505 (11)	−0.0049 (10)	0.0045 (10)	0.0007 (9)
C16	0.0526 (12)	0.0440 (10)	0.0422 (10)	0.0019 (9)	0.0055 (9)	0.0012 (8)
C17	0.0403 (10)	0.0430 (10)	0.0443 (10)	−0.0004 (8)	−0.0011 (8)	0.0036 (8)
C18	0.0530 (12)	0.0469 (11)	0.0539 (12)	0.0055 (9)	0.0062 (10)	0.0011 (10)
C19	0.0682 (14)	0.0498 (12)	0.0821 (16)	0.0095 (12)	−0.0013 (13)	−0.0096 (13)
C20	0.0693 (15)	0.0408 (11)	0.095 (2)	−0.0042 (11)	−0.0175 (15)	0.0054 (13)
C21	0.0614 (13)	0.0547 (12)	0.0736 (14)	−0.0124 (12)	−0.0097 (12)	0.0233 (12)
C22	0.0468 (11)	0.0554 (12)	0.0512 (11)	−0.0008 (10)	−0.0007 (9)	0.0098 (9)
N1	0.0454 (9)	0.0403 (8)	0.0452 (8)	0.0020 (7)	0.0056 (7)	0.0064 (7)
O1	0.0734 (10)	0.0768 (10)	0.0599 (9)	0.0050 (8)	0.0309 (8)	0.0169 (8)
O2	0.0924 (11)	0.0505 (8)	0.0466 (8)	0.0073 (7)	0.0019 (8)	−0.0101 (7)
O3	0.1119 (13)	0.0526 (8)	0.0453 (8)	0.0015 (10)	0.0186 (9)	−0.0054 (7)
S1	0.0614 (3)	0.0477 (3)	0.0389 (2)	0.0074 (2)	0.0117 (2)	0.0022 (2)

### Geometric parameters (Å, º)

**Table:**

C1—C6	1.369 (3)	C12—H12	0.9300
C1—C2	1.377 (3)	C13—C14	1.394 (3)
C1—S1	1.750 (2)	C13—H13	0.9300
C2—C3	1.373 (3)	C14—N1	1.419 (2)
C2—H2	0.9300	C15—H15A	0.9600
C3—C4	1.362 (4)	C15—H15B	0.9600
C3—H3	0.9300	C15—H15C	0.9600
C4—C5	1.363 (4)	C16—O3	1.219 (2)
C4—H4	0.9300	C16—C17	1.490 (3)
C5—C6	1.366 (3)	C17—C18	1.382 (3)
C5—H5	0.9300	C17—C22	1.383 (3)
C6—H6	0.9300	C18—C19	1.384 (3)
C7—C8	1.360 (2)	C18—H18	0.9300
C7—N1	1.420 (2)	C19—C20	1.369 (3)
C7—C15	1.483 (3)	C19—H19	0.9300
C8—C9	1.441 (3)	C20—C21	1.366 (3)
C8—C16	1.481 (2)	C20—H20	0.9300
C9—C10	1.383 (3)	C21—C22	1.385 (3)
C9—C14	1.391 (3)	C21—H21	0.9300
C10—C11	1.384 (3)	C22—H22	0.9300
C10—H10	0.9300	N1—S1	1.6701 (16)
C11—C12	1.375 (4)	O1—S1	1.4134 (15)
C11—H11	0.9300	O2—S1	1.4156 (15)
C12—C13	1.374 (3)		
			
C6—C1—C2	120.5 (2)	C9—C14—C13	121.60 (19)
C6—C1—S1	119.18 (17)	C9—C14—N1	107.17 (16)
C2—C1—S1	120.28 (17)	C13—C14—N1	131.22 (19)
C3—C2—C1	119.0 (2)	C7—C15—H15A	109.5
C3—C2—H2	120.5	C7—C15—H15B	109.5
C1—C2—H2	120.5	H15A—C15—H15B	109.5
C4—C3—C2	120.3 (2)	C7—C15—H15C	109.5
C4—C3—H3	119.9	H15A—C15—H15C	109.5
C2—C3—H3	119.9	H15B—C15—H15C	109.5
C3—C4—C5	120.5 (3)	O3—C16—C8	119.11 (16)
C3—C4—H4	119.7	O3—C16—C17	120.13 (17)
C5—C4—H4	119.7	C8—C16—C17	120.66 (16)
C4—C5—C6	120.0 (3)	C18—C17—C22	119.23 (18)
C4—C5—H5	120.0	C18—C17—C16	122.10 (17)
C6—C5—H5	120.0	C22—C17—C16	118.58 (17)
C5—C6—C1	119.7 (2)	C17—C18—C19	120.1 (2)
C5—C6—H6	120.1	C17—C18—H18	119.9
C1—C6—H6	120.1	C19—C18—H18	119.9
C8—C7—N1	107.82 (15)	C20—C19—C18	120.1 (2)
C8—C7—C15	128.60 (16)	C20—C19—H19	120.0
N1—C7—C15	123.54 (16)	C18—C19—H19	120.0
C7—C8—C9	108.87 (16)	C21—C20—C19	120.3 (2)
C7—C8—C16	128.73 (17)	C21—C20—H20	119.8
C9—C8—C16	122.38 (17)	C19—C20—H20	119.8
C10—C9—C14	119.69 (18)	C20—C21—C22	120.1 (2)
C10—C9—C8	132.63 (18)	C20—C21—H21	120.0
C14—C9—C8	107.64 (17)	C22—C21—H21	120.0
C9—C10—C11	118.9 (2)	C17—C22—C21	120.1 (2)
C9—C10—H10	120.6	C17—C22—H22	119.9
C11—C10—H10	120.6	C21—C22—H22	119.9
C12—C11—C10	120.6 (2)	C14—N1—C7	108.41 (14)
C12—C11—H11	119.7	C14—N1—S1	122.94 (12)
C10—C11—H11	119.7	C7—N1—S1	127.40 (13)
C13—C12—C11	121.9 (2)	O1—S1—O2	119.97 (10)
C13—C12—H12	119.1	O1—S1—N1	106.03 (10)
C11—C12—H12	119.1	O2—S1—N1	106.76 (8)
C12—C13—C14	117.3 (2)	O1—S1—C1	108.67 (10)
C12—C13—H13	121.4	O2—S1—C1	109.36 (10)
C14—C13—H13	121.4	N1—S1—C1	104.99 (8)
			
C6—C1—C2—C3	−0.1 (3)	C8—C16—C17—C18	−19.3 (3)
S1—C1—C2—C3	178.83 (18)	O3—C16—C17—C22	−19.3 (3)
C1—C2—C3—C4	0.1 (4)	C8—C16—C17—C22	164.22 (18)
C2—C3—C4—C5	0.8 (4)	C22—C17—C18—C19	−0.6 (3)
C3—C4—C5—C6	−1.6 (4)	C16—C17—C18—C19	−177.1 (2)
C4—C5—C6—C1	1.6 (4)	C17—C18—C19—C20	0.0 (3)
C2—C1—C6—C5	−0.8 (3)	C18—C19—C20—C21	−0.2 (4)
S1—C1—C6—C5	−179.70 (19)	C19—C20—C21—C22	1.0 (4)
N1—C7—C8—C9	−2.92 (19)	C18—C17—C22—C21	1.4 (3)
C15—C7—C8—C9	174.62 (18)	C16—C17—C22—C21	178.01 (19)
N1—C7—C8—C16	178.71 (17)	C20—C21—C22—C17	−1.6 (3)
C15—C7—C8—C16	−3.8 (3)	C9—C14—N1—C7	−1.39 (19)
C7—C8—C9—C10	−179.9 (2)	C13—C14—N1—C7	179.84 (19)
C16—C8—C9—C10	−1.4 (3)	C9—C14—N1—S1	−169.49 (12)
C7—C8—C9—C14	2.1 (2)	C13—C14—N1—S1	11.7 (3)
C16—C8—C9—C14	−179.42 (16)	C8—C7—N1—C14	2.70 (19)
C14—C9—C10—C11	−0.5 (3)	C15—C7—N1—C14	−174.99 (17)
C8—C9—C10—C11	−178.31 (19)	C8—C7—N1—S1	170.12 (13)
C9—C10—C11—C12	0.5 (3)	C15—C7—N1—S1	−7.6 (2)
C10—C11—C12—C13	−0.1 (4)	C14—N1—S1—O1	−40.28 (16)
C11—C12—C13—C14	−0.1 (3)	C7—N1—S1—O1	153.97 (15)
C10—C9—C14—C13	0.2 (3)	C14—N1—S1—O2	−169.29 (14)
C8—C9—C14—C13	178.55 (17)	C7—N1—S1—O2	24.96 (17)
C10—C9—C14—N1	−178.67 (16)	C14—N1—S1—C1	74.67 (16)
C8—C9—C14—N1	−0.37 (19)	C7—N1—S1—C1	−91.08 (16)
C12—C13—C14—C9	0.1 (3)	C6—C1—S1—O1	−159.68 (17)
C12—C13—C14—N1	178.71 (19)	C2—C1—S1—O1	21.40 (19)
C7—C8—C16—O3	137.3 (2)	C6—C1—S1—O2	−27.02 (19)
C9—C8—C16—O3	−40.9 (3)	C2—C1—S1—O2	154.07 (17)
C7—C8—C16—C17	−46.2 (3)	C6—C1—S1—N1	87.22 (18)
C9—C8—C16—C17	135.63 (18)	C2—C1—S1—N1	−91.69 (17)
O3—C16—C17—C18	157.2 (2)		

### Hydrogen-bond geometry (Å, º)

**Table:**

*D*—H···*A*	*D*—H	H···*A*	*D*···*A*	*D*—H···*A*
C15—H15*C*···O1^i^	0.96	2.59	3.525 (3)	165

Symmetry code: (i) −*x*, *y*+1/2, −*z*+3/2.
